# Endocrine modulation of stimulant use: bidirectional interactions within the hypothalamic–pituitary–gonadal axis

**DOI:** 10.3389/fnbeh.2026.1778346

**Published:** 2026-06-04

**Authors:** Sally L. Huskinson, Amy S. Kohtz

**Affiliations:** 1 Department of Psychiatry and Human Behavior, University of Mississippi Medical Center, Jackson, MS, United States; 2 Program in Neuroscience, University of Mississippi Medical Center, Jackson, MS, United States; 3 Center for Innovation and Discovery in Addictions (CIDA), University of Mississippi Medical Center, Jackson, MS, United States

**Keywords:** cocaine, endocrine dysfunction, estrous cycle, menstrual cycle, sex differences, stimulants

## Abstract

Stimulant use disorders are chronic relapsing conditions that differentially affect men and women. Growing evidence has demonstrated that endocrine systems play critical, bidirectional roles in regulating vulnerability to drug use, escalation, withdrawal, and relapse, particularly for women. In the current review we discuss the bidirectional relation between gonadal hormones; focusing on estradiol (E_2_), progesterone (P_4_), and testosterone (T); and stimulant rewards and reinforcement; focusing on cocaine, amphetamine, and methamphetamine. Most research to date has focused on cocaine and less so on amphetamine or methamphetamine. Similarly, most research has been conducted with rodents and less so with nonhuman primates and humans. Collectively, the literature supports stimulant misuse as an endocrine disorder, particularly for females, emerging from reciprocal interactions between neural and hormone systems. The reciprocal relation between gonadal hormones and stimulant rewards and reinforcement is, for the most part, reliable and robust in female rodents; however, the relation is somewhat mixed with female nonhuman primates and women. These mixed results likely are a result of the wide within- and between-subject variability in menstrual cycles (i.e., duration) and levels of ovarian hormones (e.g., E_2_ and P_4_ levels) in female nonhuman primates and women. Additionally, it is impossible to determine complete menstrual cycles prior to stimulant exposure in women as it is in female rats or nonhuman primates, though determination of menstrual cycles prior to stimulant exposure with female nonhuman primates is sparse. Female rodents, on the other hand, have much less variability in estrous cycles and corresponding E_2_ and P_4_ levels, and complete estrous cycles and hormone levels are easily obtainable prior to stimulant exposure. Finally, while the reciprocal relation between gonadal hormones and stimulants in males and men has been studied, the relation appears to be less reliably implicated in stimulant rewards and reinforcement compared with females, women, and ovarian hormones. Recognizing the hypothalamic-pituitary-gonadal (HPG) axis, including effects of stimulants on gonadal hormones and vice versa, is critical for developing individualized pharmacotherapies for stimulant misuse, particularly for women, and for understanding sex-specific stimulant-misuse phenotypes.

## 1 Introduction

Substance use disorders, including those with stimulants, are chronic, relapsing disorders characterized by compulsive or difficult to control drug-taking behavior and continued use despite negative consequences (NIDA, 2018). Through use, there are persistent alterations to neural circuits that drive rewards and stress and lead to cycles of escalating intake, short abstinence periods, and relapse, that can persist over a lifetime (Koob, 2009). Beyond the classical focus on mesolimbic rewards circuitry, endocrine systems, in particular gonadal hormones, regulate vulnerability, escalation, withdrawal, and the severity of relapse, particularly for women (Quigley et al., 2021b). Fluctuations in gonadal hormones modulate dopamine and stress pathways, effects functionally shown to impact some aspects of the rewarding and reinforcing properties of stimulants (Caine et al., 2004; Calipari et al., 2017; Chapp et al., 2024; Dreher et al., 2007; Kohtz et al., 2022; Quigley et al., 2021a). The purpose of the current review is to highlight and integrate prior research on the relation between stimulant use and endocrine function or dysfunction as well as effects of endocrine modulation of stimulant (e.g., cocaine, amphetamine, methamphetamine) rewards and reinforcement in rodents, nonhuman primates (particularly cynomolgus or rhesus macaques), and humans. We focused on procedures that measured drug rewards (conditioned place preference, subjective drug effects in humans) and reinforcement (self-administration). While prior reviews have addressed sex and hormonal influences on stimulant rewards and reinforcement, reviews that focused on the opposite relation, how stimulant misuse affects hormonal milieu, are sparse [see (Mello and Mendelson, 1997)]. In the current review, we therefore highlight the bidirectional relation between stimulant misuse and hormonal milieu, focusing on estradiol (E_2_), progesterone (P_4_), and testosterone (T).

There is large body of evidence for sex differences in stimulant misuse with clear treatment implications. While men are more likely to have a stimulant use disorder (McHugh et al., 2024), there is a greater increase in the number of new cases over the last decade in women compared to men across multiple substance classes, indicating that the gender gap in substance misuse is closing (Cornish and Prasad, 2021). Indeed, rates of stimulant use among women approached that of men for certain age groups as 12–17-years-old girls were more likely to engage in nonmedical stimulant use than their male counterparts (Nakawaki and Crano, 2012). While stimulant prescriptions were higher for men compared to women, rates of stimulant prescriptions for women have increased, and prescriptions for men have remained stable over time (Board et al., 2020). Compared to men, women experience enhanced vulnerabilities in some phenotypes associated with stimulant use [e.g., (Brady and Sinha, 2005; Sinha et al., 1999, 2000; Sinha, 2008)]. In particular, the relation between stressful life events, stress reactivity, and stimulant use disorders differs by sex (Brady and Sinha, 2005; Sinha et al., 1999, 2000; Sinha, 2008). Abundant evidence indicates that women progress more quickly from casual drug use to problematic use (McCance-Katz et al., 1999; O’Brien et al., 1992), have greater difficulty quitting (Becker and Hu, 2008; Lynch et al., 2002), and have shorter periods of abstinence than men (Griffin et al., 1989; Kosten et al., 1993). Thus, while men have higher overall rates of stimulant misuse, women experience some enhanced vulnerabilities, and the gender gap in stimulant misuse between men and women is becoming smaller.

### 1.1 Rodent sex differences in reinforcement and rewards

Similarly, sex differences in drug-seeking and -taking behavior exist preclinically in nonhuman animal models of stimulant misuse. Female rats exhibited enhanced behavioral sensitization to cocaine (Hu and Becker, 2003; Yang et al., 2007) and acquired cocaine self-administration more rapidly than males (Hu et al., 2004; Lynch and Carroll, 1999). Sex differences in acquisition of stimulant self-administration were most apparent at lower doses (in both intact and extirpated models) (Carroll et al., 2002; Hu et al., 2006; Lynch, 2006; Robinson et al., 1981), and notably, in other studies, males acquired cocaine more rapidly than females at a high dose (Kippin et al., 2005; Lynch and Carroll, 2000). Female rats also had higher intakes and motivation to earn cocaine [e.g., (Kohtz et al., 2022)], had greater cocaine-primed (Feltenstein et al., 2011; Kohtz and Aston-Jones, 2017; Kohtz et al., 2018) and stress-induced (Anker and Carroll, 2010) reinstatement, and demonstrated greater response perseveration during extinction after cocaine self-administration (Feltenstein et al., 2011; Kohtz and Aston-Jones, 2017; Kohtz et al., 2018) compared with male rats. In line with clinical research, preclinically, female rats have a heightened vulnerable phenotype when it comes to drug rewards and reinforcement compared to male rats.

### 1.2 Nonhuman primate and human sex differences in reinforcement and rewards

Compared with rodents, there are few evaluations of sex differences in the reinforcing effects of stimulants in nonhuman primates and in humans. Female cynomolgus macaques and women had higher motivation to earn cocaine compared with male cynomolgus macaques and men (Evans and Foltin, 2010; Mello et al., 2007). When choosing between oral cocaine and water, female rhesus macaques had higher cocaine intakes compared with males, particularly when females were in the follicular compared to the luteal phase of the menstrual cycle (Carroll et al., 2016). However, there also were cases in which differences between sexes did not exist for nonhuman primates and humans. When choice was between oral cocaine and saccharin, female and male rhesus macaques had similar cocaine intakes, and female intakes did not vary as a function of menstrual cycle (Carroll et al., 2016), suggesting that sex and menstrual cycle effects may differ as a function of the availability and quality of a nondrug reinforcer. Similarly, men and women self-administered cocaine at similar levels (Evans et al., 1999; Haney et al., 1998), and women self-administered more amphetamine when lower doses were available, but men self-administered more amphetamine when higher doses were available (Vansickel et al., 2010). Taken together, sex differences in the self-administration of stimulants in nonhuman primates and in humans are mixed, with sex differences emerging about as often as they do not. However, when sex differences were obtained, they tended to be in the same direction as rodents, wherein female nonhuman primates and women demonstrated the more vulnerable phenotype compared with male nonhuman primates and men.

We are unaware of any nonhuman primate studies in which rewards was assessed via conditioned place preference or other procedures. However, in humans, sex differences in subjective effects indicative of rewards following cocaine or amphetamine did not differ between men and women when menstrual cycle phase was (Collins et al., 2007) or was not controlled for (Evans et al., 1999; Haney et al., 1998; Kosten et al., 1996). Conversely, the subjective rewarding effects of d-amphetamine or cocaine were higher in men and in women in the follicular phase of the menstrual cycle compared to women in the luteal phase (Sofuoglu et al., 1999; White et al., 2002), or were higher in men compared to women when no effects of menstrual cycle phase were reported (Lukas et al., 1996). Taken together, outcomes between sexes in measures of stimulant rewards were not reliably different between men and women. In some cases, subjective rewarding effects were opposite of what would be expected if women demonstrated the more vulnerable phenotype as men often had higher subjective ratings than women. However, women in the follicular phase do appear to display a more vulnerable phenotype compared to women in the luteal phase, and this effect is consistent with measures of reinforcement (i.e., self-administration) in nonhuman primates and humans.

### 1.3 Conclusions regarding sex differences

Considering the literature reviewed across species, sex differences in stimulant use disorder and stimulant rewards and reinforcement highlights a need for gender-specific experimental and treatment approaches. This conclusion is supported by human and nonhuman animal studies showing that vulnerability can differ between sexes. Prior rodent research is generally consistent in that females demonstrate a more vulnerable phenotype compared with males. However, nonhuman primate and human research outcomes are more mixed, with sex differences appearing about as often as they do not. When sex differences did emerge with nonhuman primates and humans, they generally were in the same direction as with rodents (i.e., females demonstrated the more vulnerable phenotype compared with males), particularly for self-administration and less so with subjective drug effects where men often displayed the more vulnerable phenotype. Indeed, as noted above, men have higher overall rates of stimulant misuse compared to women. While the reciprocal relation between gonadal hormones and stimulants in males and men has been studied, the relation appears less reliably implicated in stimulant reinforcement and rewards compared with females, women, and ovarian hormones. This is discussed in more detail in subsequent sections, but it is the reason that the current review is more heavily focused on females than males. That is not to say that focusing on other reasons why males have higher rates of stimulant use compared with women are not worthy. Indeed, they are worthy, they simply are beyond the scope of the current review.

## 2 HPG axis overview and neurobiological integration

**FIGURE 1:**
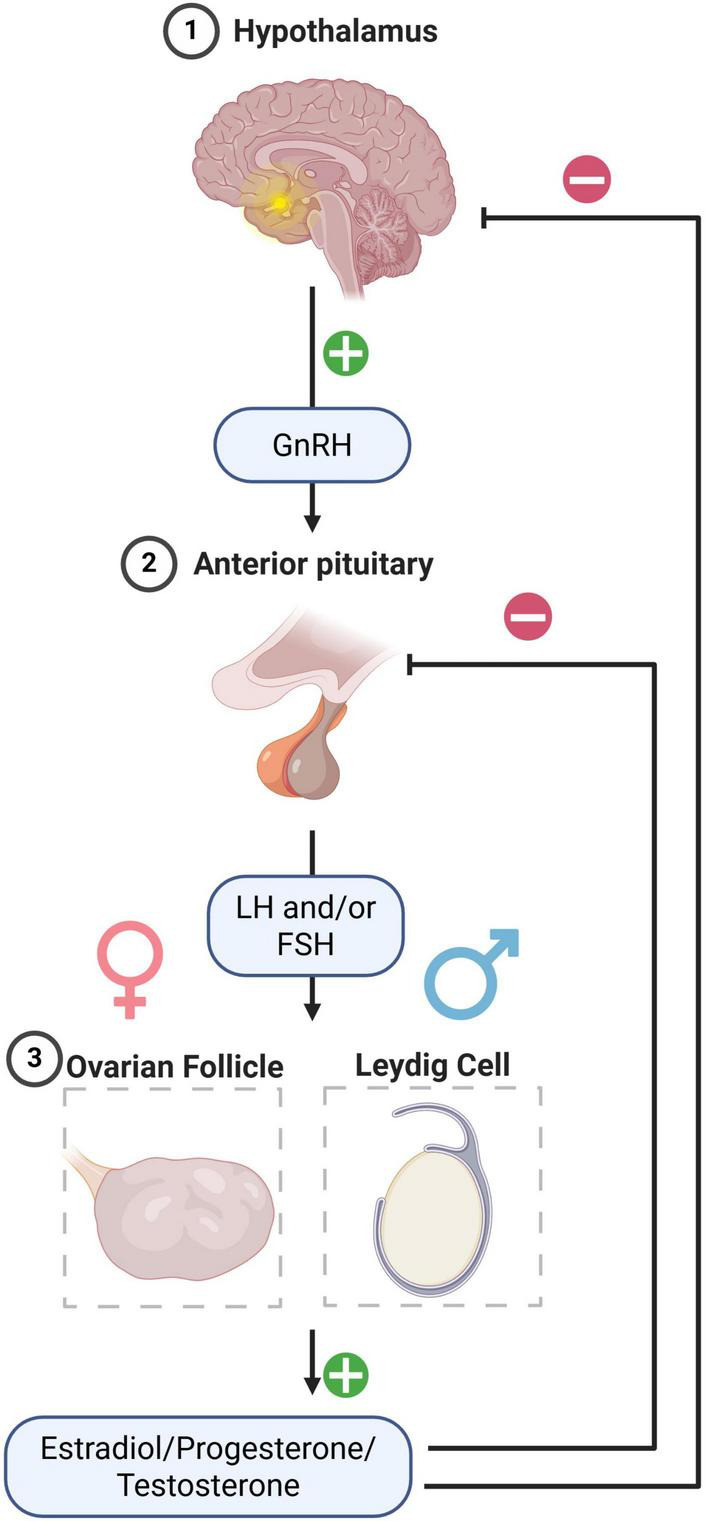
Organization and feedback regulation of the hypothalamic-pituitary-gonadal (HPG) axis. Schematic illustrating the hierarchical endocrine control of gonadal steroidogenesis in females and males. Gonadotropin-releasing hormone (gNRH) neuron in the hypothalamus **(1)** stimulate the anterior pituitary **(2)** to secrete luteinizing hormone (LH) and/or follicle-stimulating hormone (FSH). These gonadotropins act on the gonads **(3)**, promoting steroid hormone production in ovarian follicles in females and Leydig cells in males. Gonadal steroids (i.e., E_2_, P_4_, and T) exert feedback on both the hypothalamus and anterior pituitary. Symbols indicate the direction of regulation, and the diagram emphasizes conserved HPG-axis signaling across sex.

The hypothalamic-pituitary-gonadal (HPG) axis is organized as a hierarchical endocrine cascade in which gonadotropin-releasing hormone (GnRH) neurons in the hypothalamus stimulate the anterior pituitary to secrete luteinizing hormone (LH) and follicle-stimulating hormone (FSH; Figure 1). These pituitary gonadotropins then drive the gonads to produce and secrete sex steroids, including estrogens, progesterone, and androgens. Gonadal secretions of sex steroids act on widespread neural and peripheral targets to regulate a wide array of metabolic, reproductive, and psychiatric behaviors.

Centrally, sex steroids act on a broad set of neural targets relevant for rewards. Cognate and membrane steroid receptors for estrogens (Almey et al., 2015; Hwang et al., 2020; Sato et al., 2023), progesterone (Brinton et al., 2008; Frye et al., 2013, 2014; Goldsmith et al., 1997), and androgens (Dart et al., 2024; Tobiansky et al., 2018) are expressed extensively throughout the brain, including, but not limited to, regions central to rewards [Reviewed in Feltenstein and See (2008)] such as the ventral tegmental area (VTA), nucleus accumbens (NAc), prefrontal cortex (PFC), amygdala (AMYG), hypothalamus, and hippocampus (HPC). In turn, both peripherally secreted and *de novo* central production of steroids impact motivation, reinforcement learning, stress responses, and executive control [Reviewed in Ozawa (2005), Sinchak and Micevych (2001), Zheng (2009)]. In concert with peripheral effects on metabolic and autonomic systems through vagal and endocrine signals, sex steroids are a target that may be powerful modulators of drug rewards and reinforcement. Furthermore, the HPG axis could contain targets for pharmacotherapies to treat both stimulant misuse and the untoward effects that stimulant use can have on the HPG axis, particularly for females, with the latter representing a quality-of-life improvement for women with stimulant use disorders that could indirectly reduce stimulant use.

Gonadal steroid receptors modulate multiple neurotransmitter systems in both males and females, including dopaminergic, glutamatergic, and GABAergic signaling pathways, via both genomic and non-genomic mechanisms (Bendis et al., 2024). These strong interactions shape rewards processing and neural plasticity. Estrogen receptors (ERα, ERβ, GPER) rapidly modulate dopamine release, receptor sensitivity, and transporter function within mesolimbic circuits (Almey et al., 2015; Bazzett and Becker, 1994; Becker and Rudick, 1999; Bendis et al., 2024; Demotes-Mainard et al., 1990), while also influencing glutamatergic transmission by regulating AMPA/NMDA receptor trafficking (Galvin and Ninan, 2014; Smith and McMahon, 2006; Xiao et al., 2013). In parallel, E_2_ can reduce inhibitory tone (Doncheck et al., 2021; Frye et al., 1996; Krentzel et al., 2019). Androgens similarly impact dopaminergic responsivity, modulate glutamate receptor expression, and alter GABAergic inhibition, collectively affecting neuronal excitability and motivated behavior in both sexes (Frye et al., 2001; Frye, 2001, 2007; Sanchez Montoya et al., 2010; Tobiansky et al., 2018). Through these convergent mechanisms, sex-steroid signaling impacts neuromodulatory systems, shaping sex differences in stimulant rewards and reinforcement, and thereby translating to vulnerability to stimulant misuse.

## 3 Estrous and menstrual cyclicity

Estrous and menstrual cycles are characterized by species-specific patterns of gonadal hormone fluctuations that shape behavioral and physiological outcomes. While female rodents exhibit rapid estrous cycles with well-defined and stable individual stages, female nonhuman primates and women have longer menstrual cycles with extended follicular and luteal phases and substantial within- and between-subjects variability in phase duration (Harlow et al., 2000; Mihm et al., 2011; Waller et al., 1998; Wilcox et al., 2000) described in detail in section [“4.2 Nonhuman primates and humans”]. Nonetheless, across female rodents and nonhuman primates and in women, cyclic hormones emerge as critical factors that can influence behavior.

### 3.1 Hormonal cyclicity in rodents

**FIGURE 2:**
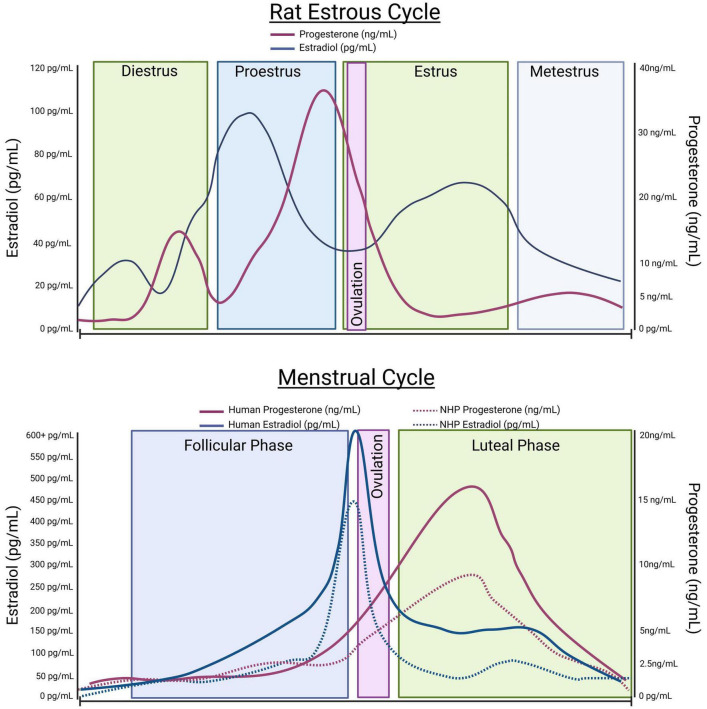
Comparative profiles of E_2_ and P_4_ across the rat estrous and nonhuman primate and human menstrual cycle. (Top) Schematic representation of circulating E_2_ (blue; pg/mL, left *y*-axis) and P_4_ (magenta; ng/mL, right *y*-axis) across the rat estrous cycle aligned to diestrus, proestrus, estrus and metestrus. Sharp rises in E_2_ occur during proestrus, and decline following ovulation, while P_4_ shows a distinct peri-ovulatory surge while remaining relatively low across the remainder of the cycle. (Bottom) Circulating E_2_ (blue; pg/mL, left *y*-axis) and P_4_ (magenta; ng/mL, right *y*-axis) across the human (solid lines) and nonhuman primate menstrual cycle aligned to the follicular and luteal phases. The follicular phase is characterized by a progressive increase in E_2_, peaking pre-ovulation; followed by a robust luteal-phase increase in P_4_, and in humans, a relatively smaller increase in E_2_. Hormone concentrations are illustrated to depict relative timing and maximal peaks reported during phases, albeit each phase has a wide range of expression.

In female rodents, the estrous cycle lasts approximately 4–5 days and consists of four stages: proestrus, estrus, metestrus, and diestrus; each associated with distinct fluctuations in circulating ovarian hormones (Long and Evans, 1922; Figure 2 Top). Unlike female nonhuman primates and women, female rats do not menstruate; instead, ovarian steroid secretion, vaginal cytology, and behavioral sexual receptivity define each cycle state (Young et al., 2005). Proestrus is characterized by a marked rise in E_2_, driven by follicular development. During proestrus, serum E_2_ reaches its peak typically ranging from 40 to 100 pg/mL, depending on strain and assay sensitivity. P_4_ rises sharply following a decline in E_2_ as the preovulatory surge in P_4_ occurs (typically ranging from 20 to 40 ng/mL), driving a subsequent surge in LH (LH not shown in Figure 2) that promotes ovulation (Smith et al., 1975). The preovulatory P_4_ surge in female rodents occurs prior to ovulation whereas with female nonhuman primates and women, the P_4_ surge occurs post ovulation (see more detail below). Estrus begins shortly following ovulation and is associated with declining E_2_ and P_4_. Metestrus follows estrus and marks the formation of early corpora lutea, during which time, E_2_ and P_4_ are low. Similarly, during diestrus, P_4_ further wanes and initiates the next follicular wave. Of note, there is intra-stage variability in E_2_ and P_4_, including the magnitude and timing of the proestrus E_2_ peak and late-proestrus P_4_ surge that contributes to variability in behavioral and physiological markers including drug-taking phenotypes. These estrous-cycle stages in female rodents are in stark contrast to gonadal hormones in male rodents, that instead fluctuate on diurnal rhythms with large individual variability (Heywood, 1980; Keating and Tcholakian, 1979). Males may show a gradual decline in T across the lifespan that coincides with reproductive decline (Kohtz and Frye, 2020). Therefore, compared to females, changes in gonadal hormones in males are relatively modest as circulating hormones in males are comparatively stable over time and do not show the pronounced cyclic fluctuations characteristic of females.

### 3.2 Nonhuman primates and humans

Female nonhuman primates, specifically rhesus and cynomolgus macaques, have a menstrual cycle that is like the menstrual cycle in women, but is markedly different from other mammals (e.g., rodents), lasting approximately 28–32 days for female macaques compared with 28–30 days in women (see Figure 2 Bottom for menstrual cycle phases and corresponding E_2_ and P_4_ levels). The menstrual cycle in female macaques begins with the first day of menses (∼5 days) starting with shedding of the uterine lining, followed by the follicular phase (∼9 days), ovulation (∼1 day or less), and the luteal phase [14–15 days; see (Weinbauer et al., 2008) for discussion]. During the early- to mid-follicular phase, E_2_ levels are relatively low (∼30–70 pg/mL), and gradually increase as FSH is released from the pituitary gland, initiating folliculogenesis which stimulates E_2_ production (Slayden et al., 2022). As the cycle progresses toward the late follicular phase (2–4 days prior to LH surges and ovulation), E_2_ rises to approximately 80–150 pg/mL, and subsequently toward a pre-ovulatory peak of >350 pg/mL. Although transiently rising, P_4_ remains low (∼0.1–1 ng/mL) until after ovulation (Slayden et al., 2022). A LH surge triggers ovulation, after which, the luteal phase begins. Within 3–8 days after ovulation, the empty follicle becomes the corpus luteum, which produces high P_4_ levels that reach their peak (3–8 ng/mL). P_4_ levels then fall sharply during the late luteal phase, prior to the beginning of menses.

The menstrual cycle in humans is similar to nonhuman primates; however, prior reports indicate a wider range of variability in the length of the follicular (10–23 days) and luteal (7–19 days) phases for women (Harlow et al., 2000; Mihm et al., 2011; Wilcox et al., 2000), with variability in the follicular phase contributing most to variations in the total length of the menstrual cycle (Waller et al., 1998). Like female nonhuman primates, E_2_ levels are relatively low at the beginning of the follicular phase and rise in the mid- to late-follicular phase. Unlike female nonhuman primates, however, E_2_ levels reach a higher peak in the follicular phase in women, particularly from the mid- to late-follicular phase [∼150–300 pg/mL; (Verdonk et al., 2019)], while P_4_ levels are low [1–2 ng/mL; (Elmlinger et al., 2002)]. Compared to macaques, women also reach higher mid-luteal peaks in P_4_, reaching 10–20 ng/mL (Anckaert et al., 2021), while E_2_ levels also remain elevated compared with female macaques, reaching ∼100–250 pg/mL (Elmlinger et al., 2002). Altogether, it is important to note that while the follicular phase is generally associated with low P_4_ and high E_2_ levels and the luteal phase is generally associated with high P_4_ levels, there are variations in E_2_ and P_4_ levels within a phase of the menstrual cycle in both women and female macaques (see Figure 2). These wide variations in P_4_ and E_2_ within phases may account for some of the inability to detect reliable sex differences or effects of menstrual-cycle phase on measures of drug rewards and reinforcement in female nonhuman primates and in women compared to the more reliable effects that tend to occur with rodents.

## 4 Stimulant-induced disruption of the HPG axis

Stimulant use can profoundly disrupt the HPG axis across species, altering circulating levels of gonadal hormones and reproductive function in both sexes. In female rodents in particular, stimulants acutely and chronically alter estrous cyclicity, hormone secretion, and reproductive behaviors. Similar disruptions are often observed in female nonhuman primates (macaques in particular) and women. These cross-species findings described below highlight the bidirectional interactions between stimulants and the HPG axis.

### 4.1 Rats

Acute and chronic exposure to cocaine can significantly disrupt the HPG axis in rodents, altering circulating gonadal hormones and disrupting estrous cyclicity. Chronic cocaine exposure disrupted gonadal steroid production and estrous cyclicity in female rats, producing acyclicity over weeks of self-administration or non-contingent exposure (Cockrell et al., 2025; Feltenstein and See, 2007; King et al., 1990, 1993; Kohtz et al., 2022, 2025). In cell culture, acute amphetamine rapidly decreased P_4_ and E_2_ release from granulosa cells, showing that amphetamine may directly impair ovarian hormone production to disrupt reproductive function (Chen et al., 2021; Kobayashi et al., 2004). Furthermore, the effects of cocaine on the female estrous cycle are reflected by dose-dependent decreases in LH and ovulation, but not prolactin or FSH (King et al., 1993), indicating selective disruption of the HPG axis with cocaine rather than global suppression of all reproductive hormones with amphetamine.

Unlike in humans or nonhuman primates, wherein hormonal state and mating behaviors are independent (Wallen and Zehr, 2004; Wallner et al., 2019), mating in rodents is tightly coupled to endocrine state. As such, hormone-sensitive behaviors (e.g., mating) in rats are commonly used as functional readouts of gonadal steroid activity and timing rather than as direct analogs of human sexual behavior. Extensive work examining endocrine disruption in rodent models therefore had relied on these established behavioral indices, which parallel underlying steroidogenesis. Therefore, we briefly include some studies in the following sections that identify their effects on hormone-dependent sexual responses, but stress that this is not relevant to human sexual behavior, *per se*. Consistent with this endocrine framework, studies have demonstrated that both estrous cyclicity and mating behaviors are strongly disrupted by stimulant exposure.

#### 4.1.1 Estrogen

The acute effects of stimulants on E_2_ are generally minimal and transient; however, acute cocaine-exposure increased central E_2_ within hypothalamic, midbrain and limbic regions in male rats at high doses (e.g., 20 mg/kg) (Kohtz et al., 2019a). The opposite effect was observed in females rats, wherein central E_2_ was decreased within hypothalamic, midbrain, and limbic regions at low and moderate doses (e.g., 5 mg/kg, or 10 mg/kg but not 20 mg/kg) (Kohtz et al., 2019b), and acute cocaine administration decreased circulating serum E_2_ levels independent of dose in females in proestrus, but not diestrus females or in males (103). Notably, in females, cocaine-induced changes in E_2_ appear to be dissociable from its effects on sexual behavior; as high dose cocaine suppressed sexual behavior in proestrus females, whereas low doses enhanced sexual behavior in diestrus females (Kohtz et al., 2010, 2019b). In males, cocaine-induced increases in central E_2_ occurred at doses that impaired copulatory behavior (Kohtz et al., 2010, 2019a), however the magnitude of E_2_ changes did not predict individual variability in sexual behavior outcomes in this study (Kohtz et al., 2019a). In contrast to cocaine, acute methamphetamine increased circulating E_2_ in female rats, concomitant with increased hormone-dependent physiological responses [e.g., vaginal lubrication (Mott and Goeders, 2023)] and hormone-dependent behavioral responses [e.g., proceptivity, mating (Rudzinskas et al., 2019)], and both effects were attenuated by ER antagonism (Mott and Goeders, 2023). Collectively, these findings indicate that cocaine and methamphetamine differentially impact peripheral E_2_ signaling and E_2_-dependent physiological responses.

Chronic psychostimulant exposure produced more persistent disruptions in ovarian function than the transient hormone fluctuations observed after acute administration. Repeated non-contingent cocaine exposure consistently disrupted estrous cyclicity, and increased tonic circulating E_2_ in female rats (Cockrell et al., 2025; King et al., 1990, 1993). Similar effects were observed with cocaine self-administration (Kohtz et al., 2022). In female rats, elevated E_2_ particularly occurs during phases when hormone levels would normally decline, suggesting that cocaine induces a tonic increase in ovarian steroid output and alters reproductive endocrine feedback (Feltenstein and See, 2007; King et al., 1990, 1993; Kohtz et al., 2022). Furthermore, cocaine-induced endocrine disruption can be attenuated by oxytocin treatment in female rats; further supporting a hypothesis of centrally mediated, neuroendocrine disruption by cocaine, rather than exclusively effects on peripheral ovarian function (Cockrell et al., 2025; Feltenstein and See, 2007; King et al., 1990, 1993; Kohtz et al., 2022, 2025).

Similar to cocaine, chronic methamphetamine exposure rapidly and persistently increased tonic E_2_ and disrupted estrus cyclicity in female rats (Mott and Goeders, 2023). These effects were observed across all stages of the estrous cycle and in fact, remained elevated after OVX, suggesting methamphetamine can amplify tonic E_2_ through both ovarian and extra-ovarian mechanisms (Mott and Goeders, 2024a). In contrast, amphetamine appears to exert an indirect effect on gonadal function. Rather than robust tonic suppression of E_2_, amphetamine exposure reduced E_2_ release from granulosa cells through disruption of cAMP and Ca2+ mediated signaling pathways, indicating impaired ovarian steroidogenesis at the cellular level (Chen et al., 2021). With the evidence that cocaine- and amphetamine-regulated transcript (CART) functions as an intraovarian regulator of follicular atresia and reproductive signaling (Chen et al., 2021; Kobayashi et al., 2004), these findings support the notion that amphetamine-related endocrine disruption may arise through altered ovarian signaling. Collectively, these studies indicate that cocaine, methamphetamine and amphetamine disrupt E_2_ regulation of the HPG axis through distinct central and ovarian mechanisms.

#### 4.1.2 Progesterone

Behavioral disruptions in hormone-dependent behaviors, like mating, parallel some of the acute cocaine-induced increases in circulating and central P_4_ within hypothalamic and limbic regions of female and male rats (Kohtz et al., 2010). As is the case with E_2_, these effects also are dependent on estrous-cycle phase in females as acute, cocaine-stimulated P_4_ secretion in the periphery and brain are greater in proestrus than in diestrus females or in male rats (Kohtz et al., 2010; Walker et al., 2001). Acute administration of high-dose cocaine disrupted P_4_-dependent mating behavior in female rats, despite increases in circulating P_4_ (Kohtz et al., 2010); however, acute, low-dose cocaine facilitated mating in diestrus females (Kohtz et al., 2010). In contrast to cocaine, acute or short-term chronic administration of amphetamine or methamphetamine facilitated P_4_-dependent female mating behaviors (Guarraci and Clark, 2003; Holder et al., 2010, 2015). Notably, effects of methamphetamine on P_4_-dependent mating were dependent on central dopamine and P_4_ (Holder et al., 2010, 2015), rather than ovarian gonadal hormones. As mentioned in prior sections, amphetamine decreased P_4_ release from granulosa cells (Chen et al., 2021), and decreased the expression of P_4_ receptors in the testis (Nudmamud-Thanoi et al., 2016), indicating there also may be direct effects of amphetamine on gonadal function and steroidogenesis. As such it is unclear if the acute effects of stimulants on female mating behaviors are dependent on disruption of ovarian P_4_, and/or through centrally mediated effects of disrupted ovarian hormones on dopamine and other rewards systems [Reviewed in Guarraci and Bolton (2014), Mott and Goeders (2024b)]. Together, these findings show that stimulants differentially modulate P_4_ and P_4_-related, hormone dependent behaviors in a dose-dependent manner, with acute cocaine generally disrupting P_4_-linked mating despite increased P_4_, while amphetamine and methamphetamine more often facilitate these behaviors. Thus, behavioral outcomes likely reflect drug-specific effects on central rewards circuitry, rather than uniform alterations in ovarian P_4_ signaling.

Chronic cocaine exposure, either contingent or non-contingent, eliminated proestrus (high P_4_) epochs, attenuated P_4_ peaks and suppressed circulating P_4_ in female rats (Cockrell et al., 2025; Feltenstein and See, 2007; King et al., 1990, 1993; Kohtz et al., 2022, 2025). Therefore, acute cocaine exposure rapidly modulated P_4_ signaling, both centrally and peripherally, indicating that P_4_ may be a neuroendocrine target of stimulant action, particularly in females. As there is observed suppression of P_4_ with chronic exposure to cocaine, these data together indicate that there is a transition from transient endocrine modulation to endocrine dysregulation with repeated stimulant exposure, particularly in females. In contrast, there are few studies to date that measure P_4_ in male or female rodents with chronic methamphetamine or amphetamine administration, even though there are studies that describe its effects on the estrous cycle (described above). There is evidence that chronic methamphetamine exposure in adolescent female mice decreased the secretion of P_4_ from granulosa cells in adulthood (Wang et al., 2016), indicating there also are long-lasting effects of chronic (meth)amphetamine on the HPG axis, like cocaine. Thus, while cocaine produces well-characterized suppression of P_4_ signaling with repeated exposure, methamphetamine and amphetamine likely also induce long-term HPG dysfunction, including impaired P_4_ synthesis and release, although these effects are less well characterized.

#### 4.1.3 Testosterone

Testosterone and androgen-dependent reproductive processes also are sensitive to stimulant exposure, with both acute and chronic exposure producing marked disruptions in androgen function. While acute, high dose cocaine stimulated neurosteroidogenesis and circulating production of T and T-metabolites (e.g., 3α-androstanediol) in both male and female rats (Kohtz et al., 2019a,b), other work demonstrated that acute, low dose or chronically administered, psychostimulants rapidly suppress circulating T and interfere with HPG regulation of GnRH release in males (Berul and Harclerode, 1989; Gordon et al., 1980; Kohtz et al., 2019a,b). Subsequent studies in males only, found that chronic administration of cocaine in utero decreases T synthesis, Leydig cell function, and disrupts androgen-dependent sexual behaviors, indicating a direct impact on both central and peripheral components of the HPG axis (Raum et al., 1990); similar effects were observed when chronic cocaine was administered to adult rats (Rodriguez et al., 1992). The effects of cocaine differ from those of amphetamines in male rats; as amphetamine and methamphetamine may instead facilitate androgen-dependent behavior in males (Fiorino and Phillips, 1999; Mihalcikova et al., 2019) and enhance incentive salience for natural and drug rewards (Frohmader et al., 2011; Kuiper et al., 2017). Together these findings indicate that although stimulants alter androgens and androgen-dependent behavior (e.g., copulation in male rodents), these effects are not causally linked. Instead, stimulant-induced disruptions of copulatory behavior in males appear downstream of dopaminergic signaling. Moreover, these effects are stimulant specific: cocaine-induced impairments may involve hormone-dependent mechanisms, whereas the effects of amphetamine and methamphetamine may be more consistent with dopaminergic sensitization in both sexes.

### 4.2 Nonhuman primates and humans

Acute and chronic exposure to stimulants (cocaine predominates the literature) also can alter circulating gonadal hormones in male and female nonhuman primates and humans and can disrupts menstrual cyclicity in female nonhuman primates (primarily macaques) and women. Acute cocaine administration stimulates release of LH and adrenocorticotropin hormone (ACTH) in intact female rhesus macaques, stimulates LH and suppresses prolactin in intact male and female rhesus macaques, and stimulates release of LH and ACTH in human men and women (Heesch et al., 1996; Loewen et al., 2017; Mello et al., 1990a,b, 1993, 1995, 2004; Mendelson et al., 1998, 2001; 2003; Teoh et al., 1994) but not in OVX female macaques (Mello et al., 1995; Sarnyai et al., 1995). Furthermore, daily cocaine self-administration in female rhesus macaques resulted in disrupted menstrual-cycle duration, amenorrhea, anovulation, and luteal-phase dysfunction (Mello and Mendelson, 1997; Mello et al., 1997). Daily noncontingent cocaine administration during days 2–14 of the follicular phase also resulted in abnormal cycle lengths (short and long) and anovulation (Potter et al., 1998, 1999).

In women, cocaine misuse can result in menstrual cycle dysfunction, including amenorrhea and luteal-phase dysfunction, resulting in compromised fertility [(Mello and Mendelson, 1997; Mello, 1998; Smith et al., 1984; Smith, 1982; Teoh et al., 1992, 1994) but see (Evans and Foltin, 2010)]; however, as noted by many of the cited authors, attributing such dysfunction to cocaine misuse alone is difficult or impossible because those who misuse cocaine also misuse a number of other drugs, including alcohol, opioids/opiates, and marijuana, all of which can disrupt reproductive function. This highlights the importance of using nonhuman animal models to determine the role of cocaine or other psychostimulants specifically on gonadal hormones. Furthermore, self-reported menstrual cycle dysfunction occurs in women who use methamphetamine (Shen et al., 2014). This study should be regarded with caution, however, as self-reported dependent measures do not always align with objective dependent measures. Furthermore, women in this study received a single blood test to determine hormone deregulations, and outcomes from a singular test should be interpreted with caution. Nonetheless, access to stimulants appears to alter menstrual-cycle function in female nonhuman primates and in women, and the effects of stimulant administration on E_2_, P_4_, and T are described in the following sections.

#### 4.2.1 Estrogen

It is possible that disruptions to the menstrual cycle result from effects of cocaine on circulating E_2_ levels. Acute administration of cocaine increased E_2_ in female rhesus macaques in the mid-follicular phase (Mello et al., 2000), an effect that also occurred most robustly in females with relatively low baseline E_2_ levels (<100 pg/ml) compared with females with relatively high baseline E2 levels [>100 pg/ml; (Mello et al., 2004)]. Conversely, acute cocaine administration did not affect E_2_ in male rhesus macaques or in mid-luteal phase female macaques (Mello et al., 2000, 2004). The latter is possibly the case because relatively high P_4_ during the mid-luteal phase may limit the ability of cocaine to increase E_2_ due to a ceiling effect (Mello et al., 2000). Importantly, differences in the ability of cocaine to increase E_2_ during the mid-follicular phase but not the mid-luteal phase or in males is likely not a result of pharmacokinetics, as plasma cocaine levels are rarely different between phases of the menstrual cycle, baseline levels of E_2_, men and women, or male and female macaques [e.g., (Collins et al., 2007; Evans and Foltin, 2004, 2006, 2010; Mello et al., 2004; Mendelson et al., 1999) but see (Lukas et al., 1996)]. Altogether, acute administration of cocaine reliably increased E_2_ during the follicular phase in female macaques but not in the luteal phase or in males. Increased E_2_ following acute cocaine administration during the follicular phase may initially contribute to menstrual-cycle dysfunction observed during chronic cocaine administration in female rhesus macaques via suppression of the release of FSH and disrupted folliculogenesis, possibly leading to anovulation and subsequent dysfunction in the luteal phase (Dierschke et al., 1985; Hutz et al., 1990; Zeleznik, 1981). Similarly, high levels of E_2_ during the early luteal phase could shorten the luteal phase and compromise fertility (Hutchison et al., 1987).

While acute cocaine administration appears to increase E_2_ in female nonhuman primates, particularly when administered in the follicular phase, chronic treatment with cocaine had opposite effects in female nonhuman primates. Chronic administration of cocaine on days 2–14 or 15 of the follicular phase reduced E_2_ and had no effects on LH (Chen et al., 2016; Potter et al., 1998). Perhaps this paradoxical effect in which chronic cocaine decreased E_2_ reflects a tolerance to acute cocaine-induced increases in E_2_. Nonetheless, chronic cocaine-induced reductions in E_2_ could contribute to menstrual-cycle dysfunction observed with chronic cocaine use in women. We are unaware of any studies in which chronic cocaine or other stimulant administration was evaluated for effects on E_2_ in male nonhuman primates or men or when administered during the luteal phase in female nonhuman primates or women.

#### 4.2.2 Progesterone

It is important to note that while acute and chronic administration of cocaine appears to have reliable effects on E_2_, there are very few indications that cocaine administration directly affects P_4_ levels in male or female nonhuman primates and humans. Indeed, acute noncontingent cocaine administration had no effects on P_4_ in female rhesus macaques during the mid-follicular or mid-luteal phase (Mello et al., 2000), and we are unaware of any studies in which P_4_ levels were evaluated after acute cocaine or other stimulant administration in males or men. However, in a study referenced above in which female rhesus macaques self-administered cocaine chronically, female subjects had several anovulatory cycles (Mello et al., 1997). Peak P_4_ levels in anovulatory menstrual cycles were reduced compared to ovulatory cycles and control animals (ranging 0.12–4.5 ng/ml), suggesting luteal-phase dysfunction in females. In this experiment, peak P_4_ levels (11.6–16 ng/ml) during ovulatory menstrual cycles while self-administering cocaine were similar to peak P_4_ levels (*M* = 15.36) in the control group. Thus, there appears to be a reduction in P_4_ levels during chronic cocaine in females only in anovulatory cycles, which are much more frequent in chronic-cocaine exposed females compared to control females.

Similarly, low P_4_ levels are observed in anovulatory cycles after cocaine is no longer available, during periods of forced abstinence, while ovulatory cycles continue to have similar peak P_4_ levels as the control group. The persistence of menstrual-cycle dysfunction into cocaine abstinence suggests that acute cocaine intoxication *per se* is not solely responsible for such dysfunction, and rather, that daily cocaine self-administration induces disruptions in the neuroendocrine regulation of the menstrual cycle (Mello et al., 1997). It also seems likely that cocaine has indirect effects on P_4_ that could result from the ability of chronic cocaine to reduce E_2_. Mello et al. (1997) did not measure E_2_, but E_2_ was measured in a similar study in which noncontingent cocaine administration also resulted in abnormally short or long cycles as well as cycles that were anovulatory (Potter et al., 1998). In the latter study, during anovulatory cycles, E_2_ failed to rise at the end of the follicular phase, FSH levels remained low, and the LH surge was blunted. Thus, anovulatory menstrual cycles in female nonhuman primates that occur following chronic cocaine administration could result from chronic cocaine-induced disruptions to the HPG axis that results in reductions in E_2_ (Chen et al., 2016; Potter et al., 1998). Low E_2_, in turn, could result in failed folliculogenesis, via a failure to trigger the LH surge (Potter et al., 1998) and thus, relatively low P_4_ levels are the result, as seen by Mello et al. (1997).

In line with P_4_ outcomes in female nonhuman primates, levels of pregnenolone (a precursor to P_4_, E_2_, and androgens) is negatively correlated with years of cocaine use in men and women who met criteria for cocaine use disorder such that lower levels predict more years of cocaine use (Milivojevic et al., 2019). Taken together, nonhuman primates and humans of both sexes with a history of chronic stimulant use do appear to have lower levels of P_4_. For females, low P_4_ levels are predominantly found during anovulatory cycles, and this effect persists into abstinence.

#### 4.2.3 Testosterone

Finally, cocaine also had acute effects on T in nonhuman primates and in humans, though effects are not consistent across studies. In male rhesus macaques, acute administration of cocaine increased T at 80 min following administration (Mello et al., 1993) but did not alter T in male rhesus macaques in a separate study (Mello et al., 2004). Conversely, acute administration of cocaine increased T in female rhesus macaques when administered in the mid- to late-follicular phase (Mott and Goeders, 2024a). Similarly, cocaine had no effects on T in men without prior drug use (Heesch et al., 1996) or in men who met criteria for cocaine use disorder (Mendelson et al., 2003). However, levels of 3α-androstanediol (testosterone-derived neuroactive steroid) are negatively correlated with years of cocaine use in men and women who met criteria for cocaine use disorder such that lower levels predicted more years of cocaine use (Milivojevic et al., 2019). This suggests an effect of chronic cocaine use on T levels in men and women, but chronic T administration preclinically has not, to our knowledge, been evaluated. Additionally, 1/3 of men with cocaine use disorder have subnormal T levels compared with men without a cocaine use disorder (Ersche et al., 2024). As with E_2_ and P_4_, chronic cocaine use may result in reduced T levels in men and women, though, the experimental studies to determine whether this occurs preclinically in nonhuman primates is sorely needed.

### 4.3 Between-species comparisons

In the following paragraphs, summaries of cross-species comparisons are provided for acute and chronic stimulant effects on E_2_, P_4_, and T. In general, far more research was conducted with rodents, highlighting a need for additional research with nonhuman primates and humans. Before summarizing these cross-species effects, several factors should be taken into consideration when making comparisons across species, particularly for female rodents, nonhuman primates, and women. Rodents have less within- and between-subject variability in E_2_ or P_4_ levels within estrous-cycle phases, whereas these levels fluctuate widely within menstrual-cycle phases and across subjects or participants. Indeed, in many of the studies with women, researchers captured snapshots of circulating hormones rather than looking at day to day correlations between behavior and circulating hormone levels. These studies would be somewhat difficult to do, but future research in this area would greatly advance the field.

#### 4.3.1 Acute effects of stimulants on E_2_, P_4_, and T

In female rodents, acute amphetamine administration reduced E_2_ release from granulosa (i.e., ovarian) cells, and acute cocaine administration, in general, reduced circulating E_2_ levels in females. This was particularly true for female rats in a relatively high E_2_ phase (i.e., proestrus) compared to those in a relatively low E_2_ phase (i.e., diestrus). Outcomes on E_2_ in female rodents with acute stimulant administration are in contrast to those obtained with female nonhuman primates as acute cocaine administration increased E_2_ in female rhesus macaques. This was particularly prevalent when baseline levels of E_2_ were relatively low compared to those with high baseline E_2_ levels or for females in the mid-follicular phase compared with the mid-luteal phase. Finally, while not assessed in female nonhuman primates, acute methamphetamine administration caused an increase in circulating E_2_ levels in female rats. Altogether, acute effects of stimulants in females are mixed depending on the type of stimulant and the species. For male rodents, administration of a relatively high dose of cocaine increased E_2_ levels, and this, too is different from nonhuman primates as acute cocaine administration had no effects on E_2_ levels in male rhesus macaques.

Similar to E_2_, acute cocaine administration in rodents generally increased P_4_ levels in female and male rats, and for females, was particularly affected during the proestrus compared to the diestrus phase. In addition, acute amphetamine administration decreased P_4_ release from granulosa (ovarian) cells and decreased P_4_ expression in the testis of male rats. Conversely, there is no evidence that acute cocaine administration affects P_4_ levels in nonhuman primates, representing another potential species difference. Additional research, including evaluation of other stimulants in nonhuman primates is needed to determine the reliability of these species differences.

Finally, acute stimulant administration was generally associated with increased T levels in male and female rats, though there were differences between cocaine and (meth)amphetamine in terms of whether altered T resulted in disfunction or facilitation, respectively, of androgen-dependent behaviors. This finding is consistent with nonhuman primates as acute cocaine administration increased T in both male and female nonhuman primates. However, there also was a separate study in which T was not altered following acute cocaine administration in male nonhuman primates. Altogether, acute effects of stimulants on E_2_ and P_4_ levels were inconsistent across species whereas outcomes with T were consistent across species as T generally was increased following acute stimulant administration.

#### 4.3.2 Chronic effects of stimulants on E_2_, P_4_, and T

While it is important to understand acute drug effects, chronic drug effects are more translationally relevant for individuals with a stimulant use disorder, because those who have a use disorder will have necessarily taken stimulants chronically. While outcomes with acute stimulant administration are somewhat inconsistent across species, chronic effects on P_4_ and T are much more consistent while E_2_ appears to diverge between rodents and nonhuman primates. Before detailing specific comparisons for E_2_, P_4_, and T, it is important to note that there is consistency across species for females in that stimulant exposure alters estrous and menstrual cyclicity, and this relation was shown in both rodents and nonhuman primates and to some extent, in women.

Chronic exposure to both cocaine and methamphetamine reliably disrupted estrous cyclicity and increased tonic circulating E_2_ in female rats. Conversely, in female nonhuman primates, chronic cocaine exposure reduced E_2_ levels, but only in anovulatory cycles which occurred on as many as 1/3 of menstrual cycles (Mello et al., 1997; Potter et al., 1999). During ovulatory cycles, E_2_ levels were unaffected.

Unlike outcomes with E_2_, chronic stimulant administration similarly blunted circulating P_4_ across species. In rodents, this was evidenced by decreased circulating P_4_, increased circulating E_2_, and an absence of proestrus, the estrous phase in which P_4_ reaches its peak. For nonhuman primates, this is evidenced by blunted E_2_ and P_4_ levels during anovulatory but not ovulatory cycles, consistent with menstrual-cycle dysfunction often reported by women with a stimulant use disorder. Additionally, the estrous- or menstrual-cycle dysfunction that is present in rodents and nonhuman primates following chronic cocaine administration persists well into abstinence, which could contribute to heightened relapse vulnerability in women.

Finally, chronic cocaine reliably reduced T levels in male rodents. This reduction in male rodents is consistent with human outcomes with T. In both men and women, 3α-androstanediol levels are negatively correlated with years of cocaine use, and a portion of men with a cocaine use disorder have relatively low levels of T compared to healthy controls. Altogether, chronic cocaine appears to reduce T levels across species, which may increase relapse vulnerability, particularly in men. Reduced T levels likely occur through secondary effects such as diminished sensitivity to natural rewards (e.g., sexual behavior) and impaired dopaminergic function rather than through a direct enhancement of drug craving; however, the impact of T decline on stimulant rewards and reinforcement remains understudied.

## 5 Gonadal hormones as modulators of stimulant use

Gonadal hormones are increasingly recognized as critical modulators involved in the progression of stimulant misuse, as fluctuations in E_2_, P_4_, and T can shape stimulant sensitivity and patterns of stimulant use. As described in detail in the prior sections, substantial hormonal variability exists within- and between-individuals and across species, adding complexity to the translation of findings from animal models to humans. Despite differences in hormonal fluctuation patterns, the modulatory roles of E_2_ and P_4_, but not T, emerge consistently in rodents and to some extent in nonhuman primates and humans, and these details are described below.

### 5.1 Rodents

#### 5.1.1 Estrogen

Across rodent models, E_2_ reliably increases multiple aspects of drug-seeking behavior. In intact cycling females, when females are in high-E_2_ estrous-cycle phases (e.g., estrus), they displayed increases in acquisition and escalation of self-administration (Hu and Becker, 2008; Hu et al., 2004), motivation (Festa et al., 2004; Niyomchai et al., 2008), and intake of stimulants (Algallal et al., 2020; Hu and Becker, 2003; Roberts et al., 1989), reflecting an effect of E_2_ to increase sensitivity to drug rewards and reinforcement. In high E_2_ cycle phases, female rats had higher progressive-ratio (PR) breakpoints (i.e., increased motivation) compared with low E_2_ cycle phases and compared to males (Roberts et al., 1989), and females earned more infusions under a PR schedule compared to males, irrespective of cycle phase (Algallal et al., 2020). Tamoxifen, an ER antagonist, inhibited acquisition of cocaine self-administration in intact females (Lynch, 2006), and either an ER antagonist or an aromatase inhibitor in males, abolished cocaine sensitization (Alvarado-Torres et al., 2023). In within-session behavioral-economics procedures, which also determine motivation to earn drug infusions as PR schedules do, females in high-E_2_ cycle phases displayed increased motivation to earn cocaine compared to females in low-E_2_ cycle phases, and males showed overall less motivation for cocaine than did females in any cycle phase (Kohtz et al., 2022, 2025). Motivation for methamphetamine did not differ based on sex, however cycle phase was not tested (Cox et al., 2013, 2017). In reinstatement tests, which are purported to measure relapse-like behavior, females in high E_2_ cycle phases had greater cue-, context- and stress-induced reinstatement with methamphetamine compared to low E_2_ phases (Cox et al., 2013). The impact of sex or endogenous E_2_ on cocaine reinstatement are inconsistent, as differing reports indicate greater or equal reinstatement in females compared to males, and minimal or inconsistent cycle phase differences (Anker and Carroll, 2010; Cason et al., 2016; Cox et al., 2013; Feltenstein et al., 2011; Kohtz and Aston-Jones, 2017; Kohtz et al., 2018; Lynch and Carroll, 2000). Other than in reinstatement, endogenous E_2_ consistently enhanced stimulant rewards and reinforcement in rodent models of both sexes.

In extirpation and replacement studies, ovariectomy (OVX) reduced drug intake and blunted self-administration of stimulants in females, an effect that was reversed by E_2_ replacement (Hu and Becker, 2003; Hu et al., 2004). In within-session behavioral-economics, OVX females with E_2_ replacement showed greater economic demand for cocaine compared to male or low-E_2_ females (Kohtz et al., 2022). In reinstatement tests, OVX in females attenuated cue-, context- and stress-induced reinstatement across stimulants, an effect reinstated by E_2_ administration (Cox et al., 2013), indicating E_2_ may prime relapse vulnerability. However, many studies indicate that E_2_ may have no effect (Caine et al., 2004; Hu et al., 2004; Lynch and Taylor, 2005) or effects at high doses only in females (Hu et al., 2004). In gonadectomized male rats, E_2_ administration increased selection for cocaine over food (Bagley et al., 2019), an effect not observed with dihydrotestosterone replacement (Menendez-Delmestre et al., 2024). Although E_2_ appears to facilitate cocaine taking in both males and females following gonadectomy, there is evidence that the underlying neural mechanisms differ between sexes, even when behavioral differences are not observed (Zhou et al., 2014). Taken together, both intact and expiration studies show that exogenous E_2_ amplifies drug rewards and reinforcement with stimulants in female and male rodents.

Estradiol profoundly shapes dopaminergic signaling through both rapid and genomic actions at ERs expressed throughout mesolimbic and nigrostriatal dopamine pathways. Early foundational studies demonstrated that E_2_ enhanced dopamine release and potentiated dopaminergic responsivity within the striatum, thereby amplifying stimulant-induced behavioral activation in both sexes (Bazzett and Becker, 1994; Becker and Rudick, 1999; Zhang et al., 2008). Subsequent work revealed that ER activation modulated catecholamine transporter function, dopamine release, D1/D2 receptor dynamics and intracellular signaling cascades (Bendis et al., 2024; Calipari et al., 2017; Chavez et al., 2010; Watson et al., 2006; Yoest et al., 2019). Parallel studies showed E_2_ enhanced dopaminergic and/or noradrenergic neuron excitability, and altered prefrontal-striatal communications in female rats, effects that may contribute to sex differences in executive function, motivation, and enhance preference or seeking for cocaine (Doncheck et al., 2021; Krentzel et al., 2019; Meyers and Kritzer, 2009; Perry et al., 2015). Multiple studies show that E_2_ potentiated the release and/or depletion of striatal dopamine in response to amphetamine (Becker, 1990; Song et al., 2019) and methamphetamine (D’Astous et al., 2004; Yu and Liao, 2000b) in females. Of note, alternative studies showed that E_2_ acts in the infralimbic cortex to facilitate extinction of cocaine seeking, likely via engagement of E_2_-dependent mnemonic mechanisms in females (Yousuf et al., 2019), indicating a sex- by region-specific involvement of E_2_ and its receptors on cocaine-seeking behavior.

#### 5.1.2 Progesterone

Progesterone is widely implicated as a protective or inhibitory modulator of stimulant-seeking behavior. In cycling females, elevated P_4_ during the estrous cycle, or that which is exogenously administered during low P_4_ cycle phases, is associated with decreased motivation for, reduced escalation of intake, reduced drug efficacy, and lower PR breakpoints for cocaine (Kerstetter et al., 2008; Kohtz et al., 2022; Niyomchai et al., 2008) and methamphetamine (Reichel et al., 2012). Exogenous P_4_ also reduced cocaine- and methamphetamine primed reinstatement and attenuated stress-induced seeking in cycling females, showing a dampening effect on relapse vulnerability (Anker and Carroll, 2010; Anker et al., 2007; Feltenstein and See, 2007; Feltenstein et al., 2011; Larson et al., 2007; Reichel et al., 2012). Exogenous P_4_ also attenuated cocaine conditioning in female, but not male, rats (Russo et al., 2003, 2010) and during a delay-discounting task, reduced “impulsive” choice for cocaine in female, but not male rats (Smethells et al., 2016). However, in delay discounting, reduced impulsive choice translates to a subject waiting for more delayed, but larger cocaine deliveries, and thus, P_4_ has the paradoxical effect of reducing impulsive choice, while also increasing total cocaine intake (Smethells et al., 2016). Finally, in OVX female rats, P_4_ has been shown to have extensive protective effects against stimulant self-administration and motivation for stimulants. P_4_ administration to OVX female rats reduced cocaine intake and escalation (Anker et al., 2012), and P_4_ can furthermore attenuate E_2_-enhanced acquisition of cocaine self-administration (Jackson et al., 2006), cocaine-seeking behavior (Anker et al., 2007), and motivation for cocaine in female rats (Kohtz et al., 2022). Together, these findings support P_4_ as a key inhibitory modulator of stimulant reinforcement in female, but not male, rodents.

Mechanistic work indicates that P_4_ and its neuroactive metabolites modulate dopaminergic and GABAergic signaling within rewards circuits, contributing to sex-specific reductions in behavioral sensitization and drug-induced locomotion for cocaine (Festa and Quinones-Jenab, 2004; Festa et al., 2004; Quinones-Jenab and Jenab, 2010; Quinones-Jenab et al., 2008; Russo et al., 2003). In contrast, in methamphetamine models, P_4_ appears to exert more direct neuroprotective effects, attenuating methamphetamine induced dopamine depletion, at least in females (D’Astous et al., 2004; Yu and Liao, 2000a). Furthermore, some studies indicate that P_4_ replacement in OVX females attenuated methamphetamine-induced synaptic plasticity dysfunction (Ghazvini et al., 2016) and striatal serotonin depletion (Yu and Liao, 2000a); however, the neuroprotective effects of P_4_ on methamphetamine-induced neurobiological outcomes were not observed in adolescent female mice (Yu et al., 2002). Thus, P_4_ generally acts as a neuroprotective modulator of stimulant-seeking behavior in females, and opposes E_2_-enhanced stimulant-seeking, possibly through modulation of dopaminergic and GABAergic rewards signaling.

#### 5.1.3 Testosterone and androgens

Androgens can impact dopaminergic signaling within mesolimbic circuits and therefore influence incentive salience and motivation for misused drugs. However, it remains unclear whether T’s modulation of rewards circuitry reflects an independent or additive effect with those of cocaine when the two are co-administered. In early studies, endogenous or exogenous T reduced acute psychomotor effects of cocaine in male rats (Long et al., 1994; vanLuijtelaar et al., 1996), however more recent studies in males show that T may rather potentiate the locomotor effects of cocaine; albeit not in a dose-dependent manner (Martinez-Sanchis et al., 2002; Minerly et al., 2008, 2010), or that T is instead essential for cocaine-sensitization (Menendez-Delmestre and Segarra, 2011). Some of these effects may be age-related, as interactions between cocaine and androgens appear stronger during adolescence compared with adulthood (Lynch, 2008; Menendez-Delmestre and Segarra, 2011; Minerly et al., 2010). Overall, T appears to modulate stimulant-related behaviors, however its role remains inconsistent; as studies show both inhibitory and facilitative effects that may depend on dose, age, and experimental context.

### 5.2 Humans and nonhuman primates

#### 5.2.1 Estrogen

There are relatively few evaluations of E_2_’s facilitative effects on stimulant reinforcement or rewards sensitivity in nonhuman primates (primarily macaques) and in humans. Also as described in the introduction, there is a failure to detect reliable differences in the rewarding or reinforcing effects of stimulants as a function of menstrual cycle phase, which suggests no effects of high vs. low levels of E_2_ on stimulant reinforcement or rewards in naturally cycling female nonhuman primates or in women (Collins et al., 2007; Cooper et al., 2013; Evans et al., 1999; Haney et al., 1998; Lukas et al., 1996). Self-administration of cocaine under a PR schedule does not differ as a function of menstrual-cycle phase with asymptotic doses (Mello et al., 2007). However, PR breakpoints are higher in the early- and mid-follicular phase compared with the late luteal phase with a dose on the ascending limb, which may suggest a role of relatively high E_2_ in the absence of P_4_ on the reinforcing effects of cocaine when relatively low doses are available, but not when relatively high doses are available. Similarly, exogenous administration of E_2_ did not reliably affect cocaine self-administration under a FR 30 schedule of reinforcement in female rhesus macaques (Mello et al., 2008). Taken together, there does not appear to be a robust demonstration in naturally cycling, female nonhuman primates or in women for a facilitative role of E_2_ in drug reinforcement.

In women, the positive subjective effects like “good drug effect” or “high,” for example, of smoked cocaine or amphetamine were higher in the follicular phase when E_2_ is relatively high, compared with the mid-luteal phase of the menstrual cycle when P_4_ is relatively high (Evans and Foltin, 2006; Evans et al., 2002; Justice and de Wit, 1999; Sofuoglu et al., 1999; White et al., 2002). Furthermore, the magnitude of good drug effects was positively correlated with salivary E_2_ levels in women (White et al., 2002). When E_2_ was systemically administered to women, it increased subjective ratings of “pleasant stimulation” but decreased ratings of “want more,” suggesting that E_2_ can enhance some of the subjective effects of amphetamine (Justice and de Wit, 2000b). In addition to evidence that supports a facilitative role of E_2_ on drug rewards, there is evidence that does not support this notion. For example, there were no differences in positive subjective drug effects (i.e., those indicative of rewards) of a single oral dose of amphetamine between the early follicular phase in women when both E_2_ and P_4_ are low compared with the late follicular phase when E_2_ is elevated but P_4_ remains low (Justice and De Wit, 2000a). Similarly, the subjective effects of intranasal or intravenous cocaine did not differ between the follicular and luteal phases in women (Collins et al., 2007; Lukas et al., 1996; Mendelson et al., 1999). Altogether, there is some evidence of a facilitative role of E_2_ in stimulant rewards for women. However, there also are some studies in which this facilitative role was not found.

#### 5.2.2 Progesterone

While it is not worth repeating all of the research described above in which cocaine rewards or reinforcement was or was not affected by cycle phase, it is important to note that when cocaine rewards or reinforcement was affected by cycle phase, it typically was blunted during the luteal phase when P_4_ levels are high (Sofuoglu et al., 1999; White et al., 2002). Similarly, exogenous P_4_ administration dose-dependently reduced cocaine self-administration under an FR schedule of reinforcement in gonadally intact and OVX female rhesus macaques without reducing food pellet self-administration (Mello et al., 2011). Exogenous administration of P_4_ (at physiological doses) to women in the follicular phase and to men also reduced the positive subjective effects (i.e., those indicative of rewards) of cocaine but did not reduce cocaine self-administration (Evans and Foltin, 2006; Reed et al., 2011; Sofuoglu et al., 2002, 2004). Similarly, in a clinical trial in post-partum women, exogenous P_4_ did not reduce the number of cocaine positive urine samples in the treatment group compared to the control group (Yonkers et al., 2014). Finally, while exogenous P_4_ did not reduce cocaine self-administration in women or reduce cocaine positive urines in a clinical trial in postpartum women, it is possible that P_4_ improves quality of life measures. Indeed, women with cocaine use disorder with relatively high circulating P_4_ levels had less anxiety and drug craving, including a lower cue-induced craving and anxiety response to a stressor (i.e., yohimbine), compared to those women with relatively low circulating P_4_ levels (Fox et al., 2008; Moran-Santa Maria et al., 2018). In cocaine-dependent men and women administered P_4_, subjects with subsequently high allopregnanolone (a P_4_ derived neuroactive steroid) levels had lower cortisol levels, a higher stress-induced cortisol response, higher positive mood scores, improved cognitive performance, and reduced cocaine cravings (Milivojevic et al., 2016). Thus, while there are mixed outcomes regarding the protective effects of P_4_ in nonhuman primates and in humans, when significant differences were obtained, they were in the direction of a protective effect, particularly for females and women, but in some cases, for males and men.

#### 5.2.3 Testosterone and androgens

In one study with nonhuman primates, T was administered prior to cocaine self-administration in female nonhuman primates. In gonadally intact and OVX female rhesus macaques, exogenous T administration dose-dependently reduced cocaine self-administration without reducing food pellet self-administration (Mello et al., 2011). We are unaware of other experiments in which T was given exogenously to male or female nonhuman primates or to humans. Perhaps because exogenous administration of T as a therapeutic is likely not a viable treatment for stimulant use disorder, given its own potential for misuse and its untoward cardiovascular effects, which could be compounded by stimulant co-administration.

#### 5.2.4 Between-species comparisons

As in prior sections, effects of gonadal hormones on stimulant rewards and reinforcement were more robust in rodents compared with nonhuman primates and humans. In female rodents, E_2_ played a reliable, facilitative role in the rewarding and reinforcing effects of stimulants whereas in female nonhuman primates and in women, E_2_ did not reliably enhance stimulant reinforcement; however, when effects were observed, they tended to be in the same direction (i.e., a facilitative role) as with rodents. When E_2_ facilitated stimulant effects similar to that observed for rodent rewards and reinforcement, E_2_ tended to do so by enhancing the subjective rewarding effects of stimulants (i.e., rewards) in women without affecting self-administration (i.e., reinforcement) in female nonhuman primates or in women. Considering obtained effects across species, E_2_ appears to drive increased drug rewards and reinforcement, particularly in female rodents and to some extent in female nonhuman primates and women.

As with E_2_, P_4_ had reliable effects on drug rewards and reinforcement in rodents, but in contrast to E_2_, P_4_ was primarily protective, blunting the rewarding or reinforcing effects of stimulants in female rodents and to some extent in male rodents. P_4_ also was protective against self-administration in some cases with female nonhuman primates, but in other cases was protective against the subjective effects of cocaine without reducing cocaine self-administration in women. While P_4_ had mixed effects on self-administration behavior, it is important to note that P_4_ did reliably improve quality of life measures in men and women (e.g., anxiety, cognitive performance, drug craving). Altogether, across species, P_4_ appears most likely to have a protective effect and to reduce stimulant rewards and reinforcement in female and male rodents, and to some extent in female and male nonhuman primates and humans.

Finally, prior evaluations of T on rewards and reinforcement are minimal across species, making cross-species comparisons difficult to make. Furthermore, exogenous administration of T as a therapeutic is not a viable treatment for stimulant use disorder given its own potential for misuse and its untoward cardiovascular effects, which could be compounded by stimulant co-administration. However, in the few experiments in which T was evaluated, it tended to enhance stimulant rewards and reinforcement in male and female rodents while reducing cocaine reinforcement in female nonhuman primates.

Given some of the identified differences across species, it is important to note again that female rodents have less within- and between-subject variability in E_2_ or P_4_ levels within estrous cycle phases, and it is relatively easy to monitor changes in fluctuating hormone levels in rodents prior to, during, and after cocaine exposure. However, E_2_ and P_4_ levels fluctuate widely within menstrual cycle phases and across female nonhuman primates or women, and it is impossible to measure fluctuating hormone levels in women prior to, during, and after cocaine exposure. While this is somewhat difficult in nonhuman primates, it is possible and should be done. Again, in many of the studies with women, researchers captured snapshots of circulating hormones to determine cycle phases alongside self-reports rather than looking at day-to-day correlations between behavior and circulating hormone levels. Similarly, while hormone levels were measured in many of the nonhuman primate studies, never were E_2_ and P_4_ levels across complete menstrual cycles determined prior to, during, and after stimulant exposure. This is important because chronic stimulant administration can alter menstrual cycles, and while relatively high or low E_2_ or P_4_ levels typically were confirmed, it is possible that in some of these cases, the menstrual cycle was disrupted, and E_2_ and P_4_ levels were blunted compared with their levels prior to stimulant exposure. This could certainly explain the mixed effects obtained with E_2_ and P_4_ on stimulant rewards and reinforcement. Future research is sorely needed in which complete menstrual cycles are determined prior to, during, and after stimulant exposure to understand the bidirectional effects of stimulants on gonadal hormones and of gonadal hormones on stimulant rewards and reinforcement. This would allow one to determine daily levels of E_2_ and P_4_ and their moment-to-moment correlation, or lack thereof, with stimulant rewards or reinforcement as well as how these hormones change over time with stimulant exposure and into abstinence.

## 6 Clinical and therapeutic implications

**FIGURE 3:**
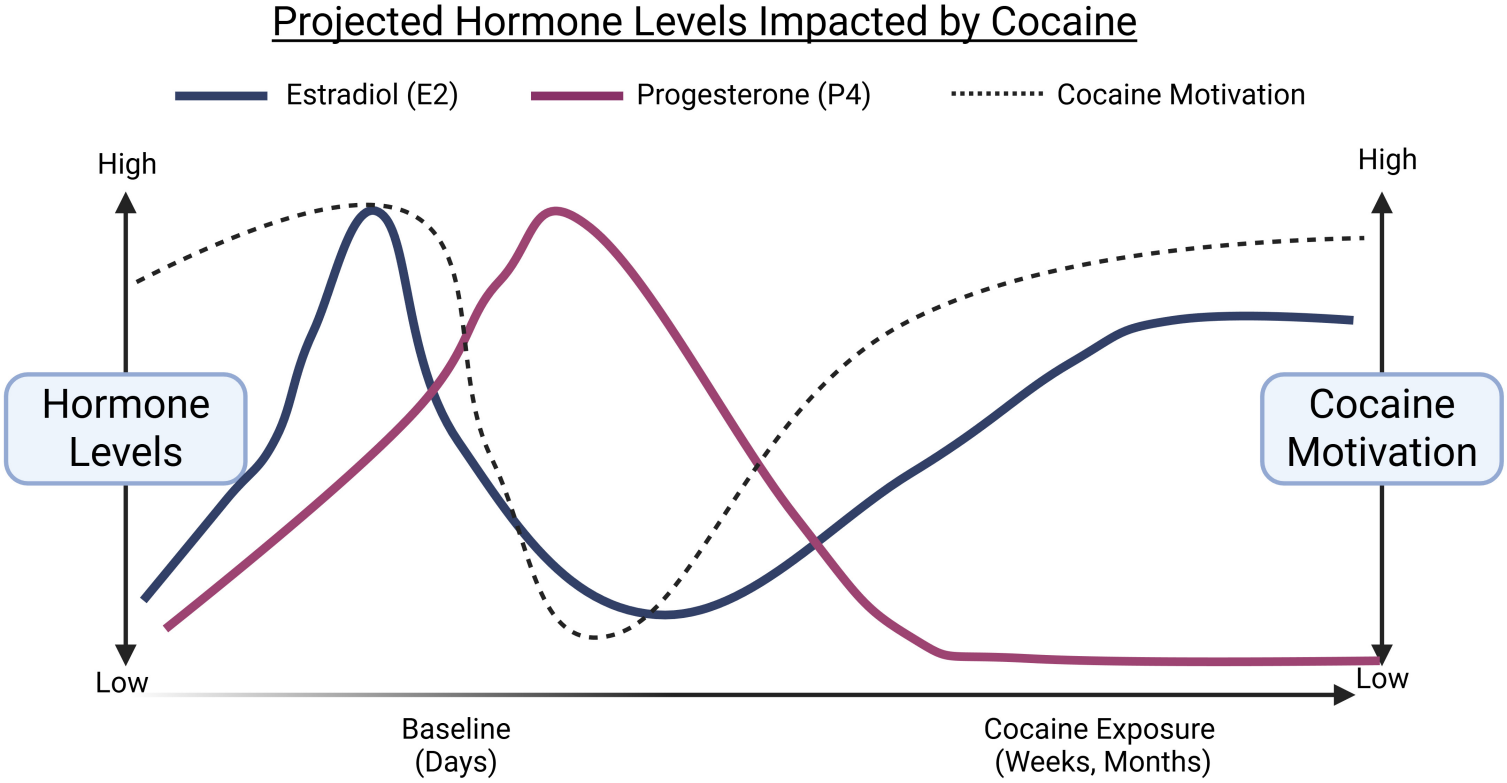
Model of cocaine-induced endocrine dysfunction in ovarian hormone dynamics and motivation for cocaine. Schematic illustrating projected changes in estradiol (E_2_; blue) and progesterone (P_4_; magenta) levels across time in relation to the motivation for cocaine over time (dotted line). During baseline period, e.g., normal cycling hormones, motivation for cocaine changes as a function of ovarian hormones. With repeated cocaine exposure (weeks in rodents, months in primates) P_4_ declines and remains suppressed, while E_2_ rises and stabilizes at moderate to high levels. In parallel, cocaine motivation progressively increases with continued exposure, reflecting enhanced drug seeking. Axes represent relative, not absolute hormone concentrations and motivational states, and depicts temporal relationships rather than measured values.

Based on all the research reviewed above, a bidirectional relation between gonadal hormones and stimulant misuse exists, implicating hormone-based therapeutics in the treatment of stimulant use disorder, particularly for females, and perhaps less so for males. Importantly, clinical translation requires distinguishing between two fundamentally different therapeutic goals: (1) hormone based-interventions aimed at directly reducing substance use and rewards-related processes, and (2) hormone treatments intended to restore or correct endocrine dysfunction that results from substance use. These goals are not necessarily interchangeable and therefore may require distinct strategies; although some of the effects of treating endocrine dysfunction resulting from substance use may in fact improve disordered substance use behavior. In fact, recent evidence in rodent models may indicate that attenuating hormonal dysfunction decreases pathological demand in females (Figure 3; Cockrell et al., 2025; Kohtz et al., 2022, 2025). While T-based treatments likely are not viable, targeting P_4_ directly or indirectly may be particularly promising for men and women. One method for treatment could be accomplished simply with exogenous administration of P_4_. This approach may be effective and preferred by some men and women with stimulant use disorders. However, if the intention of a hormone-based therapeutic is, in part or in whole, to restore normal menstrual cycles (i.e., treat the menstrual-cycle dysfunction that results from stimulant misuse), exogenous P_4_ administration will not accomplish this outcome. In rats, P4 functions as a short-lived inhibitory signal, often studied via exogenous administration. In humans, P_4_ represents a sustained counter-regulatory hormone, especially during the luteal phase. This difference likely explains why P_4_-based interventions may show stronger or more stable effects on clinically relevant outcomes in human stimulant misuse than predicted from rodent cycling alone, particularly for measures such as quality of life outcomes, despite limited effects on stimulant use. However, again, the efficacy of P_4_ to reduce addiction parameters does not extend to endocrine dysfunction, limiting its therapeutic potential. Notably, some work has shown that cocaine-induced P_4_ disruption can be attenuated by oxytocin treatment in female rats; and thus, hypothalamic peptides may be viable treatment options to regulate HPG axis function during stimulant use (Cockrell et al., 2025; Kohtz et al., 2025).

Hormonal status and contraceptive use are important variables that are under considered in clinical trials. Oral contraceptives do not alter the subjective response to acute cocaine (Kouri et al., 2002), suggesting that exogenous administration of E_2_ and P_4_ at the physiologic doses found in oral contraceptives may not be sufficient to treat cocaine use disorder. However, hormonal contraceptives are a highly heterogenous class of therapeutics, varying in hormone composition, dose, and patterns of administration; all of which may differentially influence rewards processing and treatment outcomes. While hormone contraceptive use among patients should be considered as a factor in designing treatments, it is premature to conclude that hormonal contraceptives are uniformly detrimental, effective, or ineffective in the context of stimulant use disorder treatments. Therefore, integrating reproductive health into treatments for stimulant use disorder may be essential for successful abstinence maintenance.

## 7 Conclusion

The HPG axis exerts powerful, bidirectional control over stimulant use, with these drugs both altering endocrine function and being differentially modulated by gonadal hormones. Stimulant exposure can disrupt gonadal hormone production and cyclicity in females, while endogenous and exogenous hormones modulate drug efficacy, rewards, motivation, and relapse, particularly in females. Importantly, drug-induced behavioral changes may reflect both direct pharmacological effects on neural circuits and indirect effects mediated through drug-induced changes to the HPG axis that in turn influence behavior. These reciprocal effects indicate that substance use and rewards processes are highly sensitive to the inherent cyclical dynamics of hormone release, even in cases where studies simplify these dynamics into discrete or cross-sectional comparisons. The implications of P_4_ as a protective, but disrupted, steroid hormone are clear across species, however the stimulating effects of E_2_ on stimulant misuse phenotypes are less consistent; perhaps due to extensive individual differences in nonhuman primates and humans compared to rodents. Failing to account for HPG axis function (e.g., sex, reproductive stage, contraceptive use, hormone therapy) may contribute to some variability or efficacy of hormone treatments in clinical outcomes. Therefore, incorporating endocrine assessments into clinical trial design is essential for the development of treatment strategies for stimulant use.
